# Raman spectroscopy combined with a support vector machine algorithm as a diagnostic technique for primary Sjögren’s syndrome

**DOI:** 10.1038/s41598-023-29943-9

**Published:** 2023-03-29

**Authors:** Xiaomei Chen, Xue Wu, Chen Chen, Cainan Luo, Yamei Shi, Zhengfang Li, Xiaoyi Lv, Cheng Chen, Jinmei Su, Lijun Wu

**Affiliations:** 1 Department of Rheumatology and Immunology, People’s Hospital of Xinjiang Uygur Autonomous Region, Urumqi, Xinjiang China; 2 Xinjiang Clinical Research Center for Rheumatoid Arthritis, Urumqi, Xinjiang China; 3 College of Software, Xinjiang University, Urumqi, Xinjiang China; 4 College of Software, Key Laboratory of Signal Detection and Processing, Xinjiang University, Urumqi, Xinjiang China; 5 Department of Rheumatology and Clinical Immunology, Peking Union Medical College Hospital, Chinese Academy of Medical Sciences and Peking Union Medical College, Beijing, China

**Keywords:** Classification and taxonomy, Data processing, Machine learning, Autoimmune diseases, Bioanalytical chemistry

## Abstract

The aim of this study was to explore the feasibility of Raman spectroscopy combined with computer algorithms in the diagnosis of primary Sjögren syndrome (pSS). In this study, Raman spectra of 60 serum samples were acquired from 30 patients with pSS and 30 healthy controls (HCs). The means and standard deviations of the raw spectra of patients with pSS and HCs were calculated. Spectral features were assigned based on the literature. Principal component analysis (PCA) was used to extract the spectral features. Then, a particle swarm optimization (PSO)-support vector machine (SVM) was selected as the method of parameter optimization to rapidly classify patients with pSS and HCs. In this study, the SVM algorithm was used as the classification model, and the radial basis kernel function was selected as the kernel function. In addition, the PSO algorithm was used to establish a model for the parameter optimization method. The training set and test set were randomly divided at a ratio of 7:3. After PCA dimension reduction, the specificity, sensitivity and accuracy of the PSO-SVM model were obtained, and the results were 88.89%, 100% and 94.44%, respectively. This study showed that the combination of Raman spectroscopy and a support vector machine algorithm could be used as an effective pSS diagnosis method with broad application value.

## Introduction

Primary Sjögren syndrome (pSS) is a chronic systemic autoimmune disease that primarily affects the exocrine glands, particularly the lacrimal and salivary glands, resulting in symptoms of dry eyes and dry mouth. It is sometimes accompanied by systemic features affecting extraglandular sites such as the joints, blood, kidneys, lungs, vessels, and nerves^1^. Due to its systemic involvement, pSS can present a variety of clinical manifestations that lead to confusion and delay in diagnosis. Moreover, definitive diagnosis of pSS mainly depends on the clinical manifestations, specific immunological changes, and other special examinations, such as dry eye examination, labial gland biopsy, and parotid gland tomography^2^. Early multiple examinations for pSS lead to a cumbersome diagnostic process that is costly and complex, and invasive tests such as labial gland biopsy have limitations in clinical application and are difficult to repeat. Therefore, a rapid, efficient and convenient method based on serum is more suitable.

Recently, Raman spectroscopy combined with machine learning algorithms has provided a more rapid and efficient method for the early diagnosis of many diseases^3–5^. Raman spectroscopy is an optical spectroscopic technique based on the inelastic scattering of light. It can be used to detect biological macromolecules, including proteins, lipids, and DNA, in biological samples and provides abundant molecular information at the microscopic level^6,7^. Therefore, Raman spectroscopy is commonly used in biomolecular detection. Raman spectroscopy can be used to detect changes in diseases at the biomolecular level and aid in the early diagnosis of diseases. It has been widely used in the early screening of Alzheimer's disease^8^, meningioma^9^, dengue virus infection^5^, cervical cancer^10^, oral cancers^11^, and so on.

Due to the high dimensionality of spectral data, redundant interference occurs, reducing the accuracy of the model. Therefore, we used the PCA dimensionality reduction method for feature selection to improve the accuracy of the model. This experiment was based on the serum Raman spectrum combined with the support vector machine algorithm, the radial basis kernel function was selected as the kernel function, and the PSO algorithm was the parameter optimization method. Finally, the PSO-SVM classification model was established. Through the classification results of the model, the feasibility of Raman spectroscopy combined with a support vector machine algorithm for the rapid detection of pSS patients and healthy controls (HCs) was verified.

## Methods

### Patient selection

Thirty patients with pSS who met the American–European classification criteria (AECG) and 30 healthy controls were enrolled in this study. Patients with other autoimmune diseases, malignant tumors, or active infections were excluded from this study. A signed consent form was obtained from all patients. The study was approved by the ethics committee of the People's Hospital of Xinjiang Uygur Autonomous Region.

### Sample preparation

Three milliliters of whole blood was collected into tubes without any anticoagulant and centrifuged at 1500*g* for 10 min to isolate the serum. The serum was then collected into EP tubes and frozen at − 80 °C until detection by Raman spectroscopy. For each measurement, approximately 15 µL of the serum sample was prepared in a quartz cuvette.

### Raman spectral data acquisition

All Raman spectra were recorded using a Raman spectrometer (LabRAM HR Evolution Raman Spectrometer, HORIBA Scientific Ltd.) in the range of 400–4000 cm^−1^. An Ar+ laser with a wavelength of 532 nm and power of 50 mW was used for Raman excitation. Spectra were acquired using a 10 × objective within 3 s. Three spectra per location were recorded in the wavenumber interval of 400–4000 cm^−1^. To exclude experimental interference and artifactual errors, three Raman spectra of each sample were recorded at different positions in the same plane.

### Algorithm description

Support vector machine (SVM) is a powerful supervised learning method capable of transforming data into a high-dimensional space for classification problems^12^. The SVM algorithm can be used to analyze data from small samples and data with high dimensions. It not only has a good nonlinear fitting ability and high generalization but also has the advantages of obtaining a global optimum through the objective function. At present, SVM has been widely used in the detection of diseases such as diabetes, breast cancer, and lung cancer^13–15^. In the process of SVM modeling, it is more important to choose the appropriate C and g parameters. At the same time, the application of the kernel function can also improve the performance of SVM. Separate collections. In this study, the radial basis kernel function was chosen as the kernel function.

PSO is an evolutionary computation technique for solving optimization problems^16^. The core idea of PSO is to find the optimal solution through collaboration and information sharing among individuals in the group. PSO was originally developed by Kennedy and Eberhart. It was inspired by research on bird and fish flock movement behaviors. First, a population of random particles is initialized, and then the system is updated at each iteration through searches for the optimal solution. PSO has the advantages of simple operation and a small amount of calculation, which can further reduce the time for optimizing parameters^17^. PSO-SVM has the advantages of a strong learning ability and sensitivity to small sample data and is widely used in machine learning methods. Therefore, in this study, the PSO-SVM algorithm was used to build a diagnostic model to achieve a rapid distinction between patients with pSS and HCs.

### Data analysis

Raman spectra were normalized to [0,1] by the "mapminmax" function in MATLAB r2018a. The normalization process can reduce the effect of laser power fluctuation on the sample data^18^. To improve the diagnostic accuracy and efficiency of SVM, PCA was used to characterize the serum Raman spectra.

All algorithms were implemented in MATLAB r2018a. SVM classification analysis was performed using the libsvm toolbox created by Lin and Chang.

### Informed consent

This study was approved by the ethics committee of the People's Hospital of Xinjiang Uygur Autonomous Region (in these studies). Informed consent was obtained from all participants before participating in the interview study. All methods were carried out in accordance with relevant guidelines and regulations (e.g., Helsinki guidelines).

## Results

### Spectral comparison

**Figure 1:**
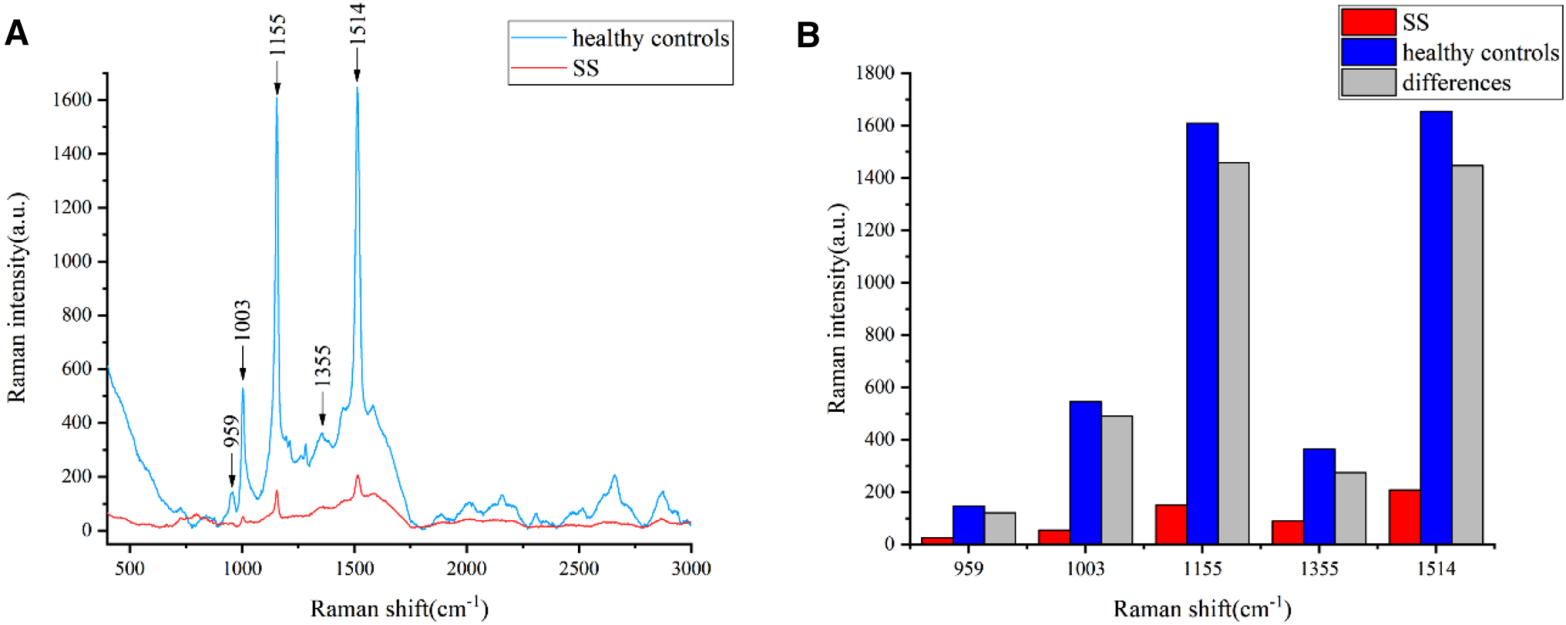
The differences in Raman spectroscopy between pSS patients and HCs. (**A**) Mean Raman spectra of HCs and pSS patients. (**B**) Major Raman peak differences between HCs and pSS patients.

The means of the raw spectra of patients with pSS and HCs were calculated (Fig. 1). A comparison of the Raman spectra showed that five peaks [proline (959 cm^−1^), phenylalanine (1003 cm^−1^), carotenoids (1155 cm^−1^), tryptophan (1355 cm^−1^), and beta-carotene (1514 cm^−1^)] were different between patients with pSS and HCs. The spectral features of these substances were assigned based on the available literature (Table 1)^19–21^. Compared to those of HCs, the Raman peak intensities of proline, phenylalanine, carotenoids, tryptophan and beta-carotene were lower in patients with pSS.

**Table 1:** Peak location of the main Raman spectra of human serum.

Peak (cm^−1^)	Assignment	Raman peak intensity comparison to healthy controls (Increase/decrease)
959	Proline	Decrease
1003	Phenylalanine	Decrease
	Phenylalanine (collagen assignment)	
1155	Carotenoids (absent in normal tissue)	Decrease
1355	Tryptophan	Decrease
1514	β-Carotene accumulation (C–C stretch mode)	Decrease

### Feature extraction

**Figure 2:**
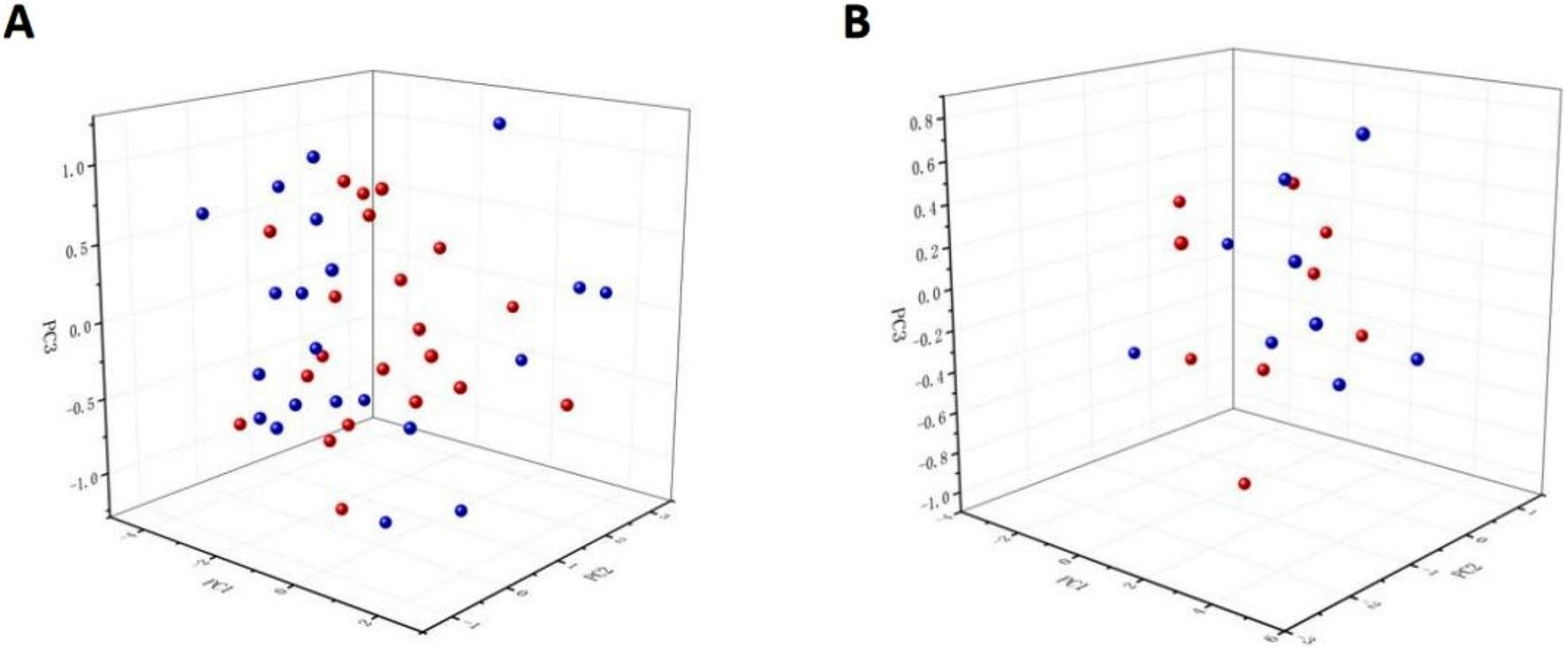
3D scatter plot of the principal components of the training and test sets. (**A**) 3D scatter plot of the principal components of the training set. (**B**) 3D scatter plot of the principal components of the test set.

After feature extraction, principal component analysis (PCA) was used for dimensionality reduction. PCs with an overall contribution of 90% will generally be retained. In this study, the total contribution of these 29 PCs was 99.99%. In addition, the most significant three PCs were extracted to plot the principal component scatter plots of the training and test sets (Fig. 2). There is a degree of variation between patients with pSS and healthy controls.

### Model evaluation

Forty-two samples (21 from patients with pSS and 21 from HCs) were randomly selected as the training set, and 18 samples (9 from patients with pSS and 9 from HCs) were selected as the test set. Classification of pSS patients and HCs was performed using an SVM classifier. In the SVM model, PSO-SVM was employed to optimize the penalty parameter C and Gaussian width g. In PSO-SVM, the search range of C was set to [2^−8^,2^8^], the search range of g was set to [2^−8^,2^8^], and the size step was set to 0.3. The PSO parameter local search ability was set to 1.5, and the overall search ability was set to 1.7; the maximum evolutionary number (MaxGen) was set to 200, and the maximum population size (sizepop) was 20. The radial basis function (RBF) was chosen as the kernel function for the SVM. The accuracy, sensitivity, and specificity of the PSO-SVM classification model were 88.89%, 100%, and 94.44%, respectively. In addition, to further illustrate the classification capability of the model, as shown in Table 2, we used the confusion matrix to evaluate the performance of the PSO-SVM algorithm.

**Table 2:** PSO-SVM model confusion matrix.

	Prediction	
	pSS	Healthy controls
True		
pSS	9	0
Healthy controls	1	8

## Discussion

pSS is a chronic systemic autoimmune disease characterized by lymphocyte proliferation and progressive exocrine gland damage^22^. Since the onset of dry syndrome is insidious, the clinical manifestations of patients are different, and the severity of the disease also varies greatly, so the early and clear diagnosis of the disease has important clinical significance to improve the prognosis of patients. However, because the pathogenesis of primary Sjögren syndrome is not yet completely clear, there is still no clear diagnostic standard, and the diagnostic standard used in clinical practice is actually a classification standard^23^. Therefore, the diagnosis of pSS needs to be confirmed by experienced specialists to prevent a large number of missed diagnoses and misdiagnoses. In addition, the main method to diagnose pSS is through a labial gland biopsy, but this method is invasive and less accepted by patients. Moreover, a labial gland biopsy has certain limitations, and the results are often inconsistent with clinical manifestations and laboratory test results in the early stage of the disease. In addition, the prevalence of pSS is as high as 3–4% in the elderly population, and these patients are often unable to tolerate a labial biopsy. Parotid angiography, parotid ultrasound and MRI are also helpful in the diagnosis of pSS^24^, but they are not included in the guidelines due to the lack of standardization of these testing techniques. Therefore, the search for new, rapid and noninvasive tests has been a hot research topic in this field.

Raman spectroscopy is a vibrational spectroscopy technique based on the Raman scattering principle^25^. Relevant studies have demonstrated the feasibility of Raman spectroscopy in different disease fields, and achieved high accuracy in many diagnoses^26–28^. Li M et al. provided a non-invasive and rapid technology for the screening of gastric cancer patients based on serum Raman spectroscopy combined with one-dimensional convolutional neural network, random forest and other machine learning methods^29^. Hyunku Shin et al. used a variety of deep learning algorithms combined with surface-enhanced Raman spectroscopy (SERS) to achieve early diagnosis of lung cancer and achieved good results^30^. Similarly, in this exploratory study, we demonstrated that Raman spectroscopy techniques combined with support vector machine algorithms can be used as an effective diagnostic method for pSS. Furthermore, we found that Raman spectroscopy can detect changes in biomolecular composition induced by pathological changes occurring between pSS and HCs, which was consistent with previous study^31^. In the experiment, due to the weak Raman signal in the detection, it is easily interfered by the fluorescent background, and the signal-to-noise ratio of the spectrum is low, which makes it difficult to distinguish different types of molecular spectral information^32,33^. Therefore, we need to use advanced pattern recognition algorithms to improve the classification accuracy. Principal component analysis (PCA), an unsupervised feature extraction algorithm that can reduce the dimensionality of Raman spectral data^34^, has been widely used by many researchers for the extraction of Raman spectral features. Similarly, pattern recognition requires powerful classifiers. In recent years, support vector machine (SVM) have been widely used in the field of pattern recognition with obvious effects. In this study, particle swarm optimization (PSO)-SVM was selected as the method of parameter optimization to rapidly classify patients with pSS and healthy controls.

In this study, we found some differences in the Raman spectra of serum from patients with pSS and HCs. Compared to those in HCs, the proline, carotenoids, and tryptophan peaks were of lower intensity in patients with pSS. This may indicate that pSS patients experience metabolic changes that result in less proline, carotenoids, and tryptophan than HCs. Studies have shown that metabolic levels of proline and tryptophan are significantly altered by the effects of pSS^35^. And carotenoids can be converted into vitamin A^36^, which in appropriate concentrations can in turn improve the immune function of cells^37^. The main etiology of pSS is associated with abnormal immune function^38^, which represents a possible deficiency of vitamin A in patients with pSS. Based on the differences in serum spectra, the accuracy rate of the PSO-SVM classification model reached 94.44%. Thus, it can be used to rapidly discriminate patients with pSS and HCs. As there was a limited sample size in this study, we plan to collect more samples to validate the results of this exploratory experiment in the future and evaluate the effect of serum Raman spectroscopy for pSS diagnosis.

## Conclusion

In this study, we used Raman spectroscopy combined with the PSO-SVM algorithm to rapidly diagnose pSS based on serum samples obtained from pSS patients and healthy controls. The spectral data were reduced using PCA, and the first 29 PCs were taken as input. Through the evaluation metrics of the model, we found that PSO-SVM performed stably, with model specificity, sensitivity and accuracy results of 88.89%, 100% and 94.44%, respectively. This study showed that Raman spectroscopy combined with a support vector machine algorithm could be used as an effective pSS diagnosis method.

## Data Availability

The datasets generated and analyzed during the current study are not publicly available due to data privacy laws but are available from the corresponding author on reasonable request.
